# Biological occupational allergy: Protein microarray for the study of laboratory animal allergy (LAA)

**DOI:** 10.3934/publichealth.2018.4.352

**Published:** 2018-10-09

**Authors:** Maria C D'Ovidio, Annarita Wirz, Danila Zennaro, Stefania Massari, Paola Melis, Vittoria M Peri, Chiara Rafaiani, Maria C Riviello, Adriano Mari

**Affiliations:** 1 Department of Occupational and Environmental Medicine, Epidemiology and Hygiene, Italian Workers' Compensation Authority (INAIL), Monte Porzio Catone (Rome), Italy; 2 Santa Lucia Foundation (FSL), Rome, Italy; 3 Associated Centers for Molecular Allergology, Rome, Italy; Center for Molecular Allergology, IDI-IRCCS, Rome, Italy; 4 Department of Occupational and Environmental Medicine, Epidemiology and Hygiene, Italian Workers' Compensation Authority (INAIL), Rome, Italy; 5 Italian National Agency for New Technologies, Energy and Sustainable Economic Development (ENEA), Rome, Italy; 6 Cellular Biology and Neurobiology Institute, National Research Council of Italy (CNR), Rome, Italy

**Keywords:** occupational allergy, laboratory animals, protein microarray, questionnaire, health workers

## Abstract

**Background:**

Laboratory Animal Allergy (LAA) has been considered a risk for the workers since 1989 by the NIOSH. About one third of the Laboratory Animal Workers (LAWs) can manifest symptoms to LAA as asthma, rhinitis, conjunctivitis and cutaneous reactions. The prevalence of LAA-induced clinical symptoms has been estimated with a great variability (4–44%) also due to the different methodologies applied.

**Objective:**

Evaluate the prevalence of IgE positivity to mouse and rat allergens in LAWs and assess which factors are predisposing to sensitization among subjects exposed to laboratory animals in the workplace.

**Methods:**

One hundred LAWs were invited to fill out a questionnaire regarding current allergic symptoms, atopic history, home environment, previous and current occupational history. IgE reactivity *versus* specific allergens was evaluated with ImmunoCAP ISAC.

**Results:**

Out of one hundred LAWs, 18% had a serum susceptibility to mouse and/or rat allergens and 42% reported to have occupational allergy symptoms. Combining the results acquired by ImmunoCAP ISAC and questionnaire, 17% of LAWs have been defined as LAWs-LAA positive since they present a positive IgE response and allergy symptoms, 1% LAWs-LAA sensitized, 25% LAWs-LAA symptomatic and 57% LAWs-LAA negative. Presence of previous allergy symptoms in work and life environment were significantly related to LAWs-LAA positive/sensitized.

**Conclusions:**

The study aimed to define the immunological profile of LAWs using the proteomic array as an innovative approach in the study of environmental and occupational exposure to allergens. We suggested a definition of LAWs-LAA considering serum IgE response and presence of allergy symptoms. The proposed approach has the advantage to provide a standard methodology for evaluating the specific IgE responsiveness to animal allergens in specific workplace also considering the immunological profile of workers referred to exposure in life and occupational environment.

## 1. Introduction

Occupational allergy diseases result from a complex interaction between endogenous and exogenous factors, as well as individual susceptibility and level of exposure to allergens both in work and life environment. Clinical symptoms can be respiratory, ocular, cutaneous or systemic resulting in asthma, rhinitis, conjunctivitis, dermatitis or systemic diseases. Numerous substances commonly used at work can be responsible for several allergic conditions; high-molecular-weight (glyco) proteins—vegetable and animal—and low-molecular-weight chemical substances can be able to induce occupational asthma (OA), occupational rhinitis (OR), conjunctivitis and cutaneous reactions in sensitized workers. OA represents about the 25% of respiratory diseases in the workplace and it has been evaluated that a 17% of asthma in adults is due to occupational exposures as reported by NIOSH (https://www.cdc.gov/niosh/topics/asthma/epidemiology.html). Moreover, prospective cohort studies reported an incidence rate of 0.2–3.5 per 100 person/year in workers exposed to laboratory animals [1]–[3]. Estimates of the incidence of OA in the general population ranged from 17 to 174 new cases per million active workers per year, suggesting an underestimation in most countries and the need for early diagnosis, identification of exposure agents, treatment and management of this disease [1],[3],[4].

Laboratory Animal Allergy (LAA) has been considered a risk for the workers by the National Institute for Occupational Safety and Health (NIOSH) in 1998 in the USA [5]–[7]. The sensitization generally occurs within the first three years of employment following exposure mainly to mice and rat urinary proteins [8],[9]. It has been reported that about one third of the Laboratory Animal Workers (here identified as LAWs) [7] can manifest symptoms to LAA; the prevalence of LAA-induced clinical symptoms in LAWs has been estimated with a great variability in terms of percentages (also due to the different methodologies applied) passing from 4–22% to 11–44% [10]–[16].

The onset of symptoms—since the beginning of exposure—seems to occur within the first three years of work [11],[17],[18]. The most important and commonly reported disease for LAA is asthma that may develop in one out of ten workers exposed to rats [19],[20]. The inhalation is the main route of exposure to animal allergens; in addition to eye contact, direct skin contact, animal bites, needle-stick injuries with a contaminated needle containing animal allergens [21].

Although the LAA is referred to all laboratory animals, rats and mice are those mainly responsible for LAA not because they have more allergic power but because they are more diffusely used in biomedical research in Europe [22]. Mice and rat urines represent the main source of allergens though other sources of rodent allergens are hair, dander, saliva and serum [23]. For different laboratory animal species such as rabbit, hair and saliva are the principal sources of allergens [24]. Several laboratory animals' allergens belong to lipocalins, small proteins involved in the transport of some hydrophobic molecules with low molecular weight, such as urinary odorants of rodents functioning as pheromones or pheromone-binding proteins [25]–[28]. Some allergens of the laboratory rodents have been described in the literature—Mus m 1, Mus m 2, Rat n 1, and Rat n 4 for mouse and/or rat respectively are the most cited [9],[25],[29],[30].

The study is aimed to further investigate characteristics of LAWs in relation to allergy symptoms and serum IgE response and evaluate the effect of occupational exposure in animal facilities in terms of immunological sensitization.

## 2. Materials and method

The study was designed with a multidisciplinary approach (as a collaboration between biologists, physicians and statistics competent in the areas of animal facility, clinical allergy, molecular allergy, occupational medicine and epidemiology) through a specific questionnaire that takes into consideration the LAWs' clinical history, previous and actual occupational exposure to laboratory animals, exposure to animals in living environments, physical examination and serological evaluation of IgE positivity with proteomic array.

### 2.1. Laboratory animal workers (LAWs)

The study was approved by the Institutional Ethical Committee of the Istituto Dermopatico dell'Immacolata, Rome. All subjects signed an informed consent form when undergoing all tests. In one hundred LAWs the susceptibility to allergens of animal and vegetable, food allergens, and other was estimated using the protein microarray [31].

All workers were invited to fill out a questionnaire on current allergic symptoms, atopic history, home environment, previous and current occupational history, job description. The personnel involved in the care and use of animals were scientists, animal technicians, veterinarians working in different animal facilities.

The questionnaire used in this study was drawn from the literature [32] and subjected to a validation process through the following steps: (a) translation from English into Italian made by the researchers; (b) translation from Italian to English done by a native speaker; (c) comparison of English and Italian versions not included in the group of 100 LAWs studied; (d) assessment of the problems that emerged from the individual questions; (e) elaboration of the definitive questionnaire used and administered to 100 LAWs. Validation is a process that is still in place to assess the reliability of an instrument such as the clinical-anamnestic questionnaire [33].

### 2.2. Multiplexing IgE detection

Specific IgE for allergenic molecules were detected by ISAC 103 microarray test (Phadia Multiplexing Diagnostics, Vienna, Austria) including the ISAC microarray system, all allergens and reagents, the spotted microarray slides, the software, and performed as previously reported [34]. The testing procedures have been carried out following the manufacturer's instructions. A customized version of the ISAC microarray (ISAC Exp96) has been developed to carry out the characterization of the IgE reactivity towards the new allergens not available on the commercial ISAC 103 allowing to identify mouse (Mus m 1, Mus m 4) and rat (Rat n 4) allergens. Details on biochemical, immunological, and clinical features of each allergen are available via the Allergome website (www.allergome.org) [35]. Biochips with different number of spotted allergens have been used during this study; both ISAC 103 and Exp96 IgE testing were performed as previously reported [36].

### 2.3. Data storing, processing and statistics

All data obtained by questionnaire and by microarray test have been used to create a single database. Statistical analysis was carried out with IBM SPSS software (version 22) and a criterion of probability value of *p* < 0.05 was defined. Crude prevalence ratios were calculated according to the LAWs-LAA grouped by positive, sensitized, symptomatic and negative and differences in categorical study variables were evaluated by using chi-squared test. Prevalence ratios (PRs) were calculated by multiple logistic regression analysis in order to determine the relationship between risk factors and the serological susceptibility.

## 3. Results

Study population is represented by 72 females (mean age 36.5 ± 8.05 std dev) and 28 males (mean age 37.9 ± 8.20) with a working history of 8.32 years (std dev = 6.55) and 6.31 years (std dev = 5.372) respectively.

**Figure 1.**
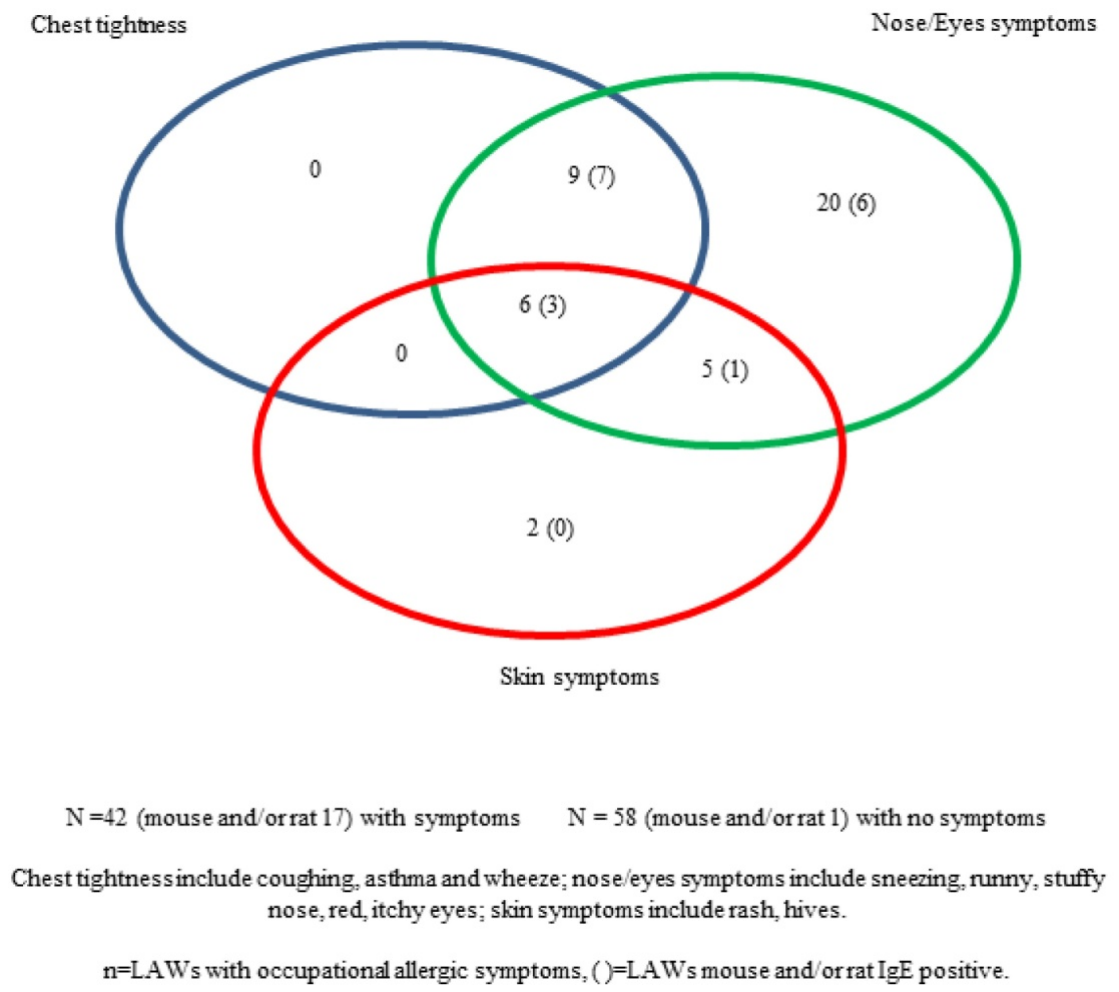
Prevalence of reported allergy symptoms among 100 LAWs.

According to information acquired by questionnaire, allergic symptoms were highly prevalent in the study group: 71% of LAWs declared to have had allergic symptoms/to be symptomatic; 12% reported to be allergic to pet animals. Regarding occupational exposure refers to workers handling with laboratory animals, 49% reported a previous occupational exposure with laboratory animals and 92% of them with mouse and/or rat. Among 100 LAWs, 9% reported to have had allergic symptoms to laboratory animals but only 8% resulted to be mouse and/or rat positive. Current professional exposure to laboratory animals was reported by 97% of workers and 93% of them handled with mouse and/or rat. A percentage of 42% of LAWs presented occupational allergic symptoms, following exposure to source in workplace, which occur mainly (26%) within the first three years of work. Most of the reported symptoms were nose symptoms such as sneezing, runny and stuffy nose for 40% workers, red and itchy eyes for 26% workers; 15% reported chest tightness including coughing, asthma and wheeze and 13% skin symptoms including rash, hives (Figure 1).

**Figure 2.**
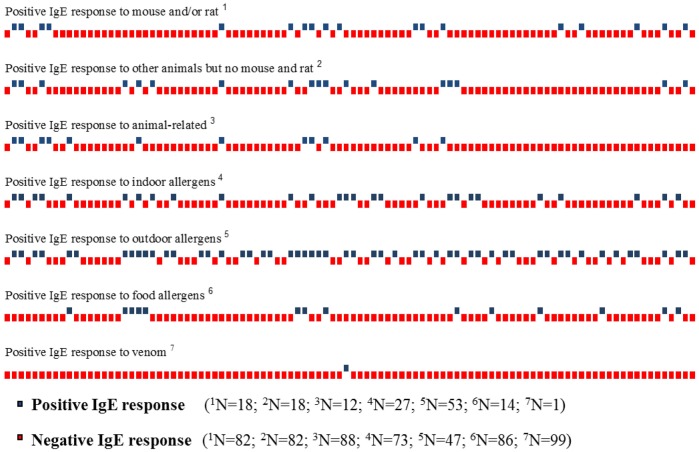
Positive and negative IgE response to allergens for each of 100 LAWs.

**Figure 3.**
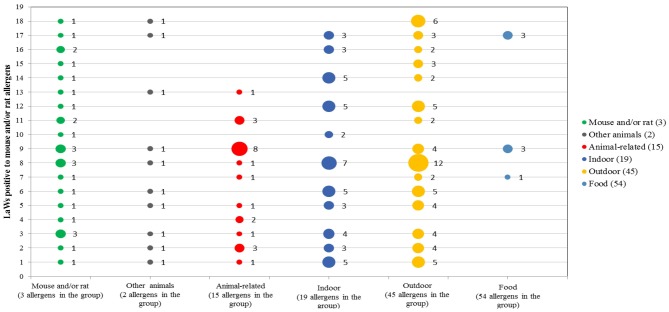
Immunological profile of 18 LAWs positive to mouse and/or rat allergens by grouped class of allergens (Numbers next to the points represent the sum of allergens in each grouped class referred to serum IgE positivity).

Multiplexing IgE detection on 100 LAWs showed the presence of IgE against food allergens, aeroallergens (vegetables and animals) and other allergens. All allergens were grouped in five subclasses as indicated: Mouse and/or rat and their related, other animals, indoor, outdoor, food and venom (Figure 2). Some of the animal allergens tested were mouse, rat, cat, dog and horse; 18 LAWs were positive for Mus m 1 or Mus m 4 or Rat n 4 animal allergens. The immunological profile of the 18 LAWs IgE positive to mouse and/or rat allergens is represented in Figure 3.

According to the positivity of specific serum IgE laboratory animal allergens and the presence of allergy symptoms the distribution of 100 LAWs is showed in Table 1.

**Table 1.** Distribution of 100 LAWs-LAA considering serum IgE* response and allergy symptoms**.

LAWs-LAA	Serum IgE + Allergy symptoms +	Serum IgE + Allergy symptoms −	Serum IgE − Allergy symptoms +	Serum IgE − Allergy symptoms −
LAWs-LAA positive	17			
LAWs-LAA sensitized		1		
LAWs-LAA symptomatic			25	
LAWs-LAA negative				57

*Mouse and/or rat IgE response determined by protein microarray. **Actual occupational allergy symptoms referred in the questionnaire.

Crude prevalence ratios were calculated and statistical significance between studied variables has been evaluated for the LAWs-LAA (Table 2). In this study, we choice to give a central role to serological IgE positivity to mouse and/or rat allergens and on this assumption we group together positive and sensitized category since the second category consists of only one worker. The comparison between LAWs-LAA positive/sensitized and LAWs-LAA symptomatic respect to LAWs-LAA negative showed a statistically significant association with the presence of experienced allergy symptoms, personal history or domestic animal or previous occupational allergy, allergy symptoms working with animals.

**Table 2.** Characteristics of LAWs according to serum IgE response and allergy symptoms (N = 100).

Variable	LAWs-LAA positive/sensitized^1^	LAWS-LAA symptomatic^2^	LAWS-LAA negative^3^	*p* value	
	N (%)	N (%)	N (%)	*	#
Age in year, mean (std)	37.2 (8.14)	36.6 (8.60)	36.1 (7.49)		
Gender					
Male	6 (33.3%)	7 (28.0%)	15 (26.3%)	0.563	0.874
Female	12 (66.7%)	18 (72.0%)	42 (73.7%)		
Age class					
20–30	5 (27.8%)	7 (28.0%)	16 (28.1%)	0.405	0.911
31–40	10 (55.6%)	9 (36.0%)	23 (40.4%)		
41+	3 (16.7%)	9 (36.0%)	18 (31.6%)		
Experienced allergy symptoms					
Yes	17 (94.4%)	24 (96.0%)	30 (52.6%)	0.001	0.000
No	1 (5.6%)	1 (4.0%)	27 (47.4%)		
Personal history allergy					
Yes	16 (88.9%)	19 (76.0%)	30 (52.6%)	0.006	0.047
No	2 (11.1%)	6 (24.0%)	27 (47.4%)		
Domestic animal allergy					
Yes	4 (22.2%)	6 (24.0%)	2 (3.5%)	0.011	0.004
No	14 (77.8%)	19 (76.0%)	55 (96.5%)		
Previous occupational allergy					
Yes	3 (16.7%)	5 (20.0%)	1 (1.8%)	0.014	0.003
No	15 (83.3%)	20 (80.0%)	56 (98.2%)		
Allergy to mouse and rat					
Yes	3 (16.7%)	5 (20.0%)	0 (0%)	0.002	0.000
No	15 (83.38%)	20 (80.0%)	57 (100.0%)		
Actual occupational allergy					
Yes	17 (40.5%)	25 (59.5%)	0 (0.0%)	0.000	0.000
No	1 (1.7%)	0 (0.0%)	57 (98.3%)		
Occupational exposure (year)					
0–3	6 (15.8%)	9 (23.7%)	23 (60.5%)	0.291	0.550
4–7	4 (21.1%)	3 (15.8%)	12 (63.2%)		
8–11	3 (18.8%)	6 (37.5%)	7 (43.8%)		
12–15	0 (0.0%)	5 (38.5%)	8 (61.5%)		
16+	5 (35.7%)	2 (14.3%)	7 (50.0%)		
Nose/eyes symptom working with animals					
Yes	17 (94.4%)	23 (92.0%)	0 (0%)	0.000	0.000
No	1 (5.6%)	2 (8.0%)	57 (100.0%)		
Asthma/chest tightness working with animals					
Yes	10 (55.6%)	5 (20.0%)	0 (0%)	0.000	0.000
No	8 (44.4%)	20 (80.0%)	57 (100.0%)		
Skin symptoms working with animals					
Yes	4 (22.2%)	9 (36.0%)	0 (0%)	0.000	0.000
No	14 (77.8%)	16 (64.0%)	57 (100.0%)		
Time of onset of allergic symptoms					
< 10 minutes	3 (16.7%)	9 (36.0%)	57 (100.0%)	0.000	0.000
> 10 minutes	15 (83.3%)	16 (64.0%)	0 (0%)		
Positive to animals but not mouse or rat					
Yes	10 (55.6%)	3 (12.0%)	5 (8.8%)	0.000	0.650
No	8 (44.4%)	22 (88.0%)	52 (91.2%)		
Positive indoor					
Yes	11 (61.1%)	7 (28.0%)	9 (15.8%)	0.000	0.199
No	7 (38.9%)	18 (72.0%)	48 (84.2%)		
Positive outdoor					
Yes	15 (83.3%)	16 (64.0%)	22 (38.6%)	0.001	0.034
No	3 (16.7%)	9 (36.0%)	35 (61.4%)		
Positive food					
Yes	3 (16.7%)	5 (20.0%)	6 (10.5%)	0.485	0.247
No	15 (83.3%)	20 (80.0%)	51 (89.5%)		
Positive rat or mouse					
Yes	18 (100.0%)	0 (0.0%)	0 (0.0%)	0.000	-
No	0 (0.0%)	25 (100.0%)	57 (100.0%)		
Total	18 (18.0%)	25 (25.0%)	57(57.0%)		

^1^LAWs-LAA positive/sensitized: Positive IgE response with and without allergy symptoms.

^2^LAWs-LAA symptomatic: Negative IgE response with allergy symptoms.

^3^LAWs-LAA negative: Negative IgE response without allergy symptoms.

*LAWs with LAA positive/sensitized vs. LAA negative.

#LAWs with LAA symptomatic vs. LAA negative.

Positivity to indoor, outdoor and animal allergens other than mouse and/or rat allergens were significantly related to LAWs-LAA positive/sensitized while actual occupational allergy is significantly related to LAWs-LAA symptomatics.

Prevalence ratios were presented in Table 3 for the studied variables with respect to the control group (LAWs-LAA negative). Factors which are more likely to induce LAA positivity/sensitized were the presence of previous allergy in life and working environment, specifically: Domestic animal allergy (PRs = 3.286; 95% CI = 1.577–6.846), previous occupational allergy (PRs = 3.550; 95% CI = 1.724–7.312), allergy to mouse and/or rat (PRs = 4.800; 95% CI = 3.060–7.530), allergic symptoms working with animals (asthma/chest tightness PRs = 8.125; 95% CI = 4.246–15.547; skin PRs = 5.071; 95% CI = 3.172–8.109), positivity to animals but not mouse or rat (PRs = 5.000; 95% CI = 2.391–10.456), indoor (PRs = 4.321; 95% CI = 1.947–9.594), outdoor (PRs = 5.135; 95% CI = 1.620–16.282).

Concerning the type of activity carried out with animals, receiving animals or working into the reproduction room or feeding or administering substances are more likely to be a LAWs-LAA symptomatic than LAWs-LAA negative (PRs = 2.038; 95% CI = 1.051–3.954; PRs = 1.988; 95% CI = 1.057–3.740; PRs = 2.227; 95% CI = 1.143–4.341).

**Table 3.** Prevalence ratios (PRs) of LAWs-LAA positive/sensitized and LAWs-LAA symptomatic compared to LAWs-LAA negative.

Variable	LAWs-LAA positive/sensitized^1^		LAWs-LAA symptomatic^2^	
	PRs	95% CI	PRs	95% CI
Experienced allergy symptoms	10.128	1.424–72.026	12.444	1.775–87.259
Personal history allergy	5.043	1.250–20.342	2.133	0.954–4.769
Domestic animal allergy	3.286	1.577–6.846	2.921	1.673–5.099
Previous occupational allergy	3.550	1.724–7.312	3.167	1.884–5.322
Allergy to mouse and rat	4.800	3.060–7.530	3.850	2.641–5.613
Asthma/chest tightness working with animals	8.125	4.246–15.547	3.850	2.641–5.613
Skin symptoms working with animals	5.071	3.172–8.109	4.563	2.959–7.035
Positive to animals but not mouse or rat	5.000	2.391–10.456	1.261	0.483–3.297
Positive indoor	4.321	1.947–9.594	1.604	0.812–3.170
Positive outdoor	5.135	1.620–16.282	2.058	1.031–4.111
Receiving animals	1.675	0.616–4.551	2.038	1.051–3.954
Reproduction room	0.682	0.224–2.082	1.988	1.057–3.740
Receiving animal room	1.300	0.457–3.699	1.676	0.832–3.378
Feeding or administering substances	1.063	0.453–2.490	2.227	1.143–4.341

^1^LAWs-LAA positive/sensitized: Positive IgE response with and without allergy symptoms.

^2^LAWs-LAA symptomatic: Negative IgE response with allergy symptoms.

## 4. Discussion

Our study evaluated, for the first time, the risk of exposure to animal laboratory allergens in animal facilities with a proteomic-based allergy evaluation through a multidisciplinary approach addressed to identify: Immunological profile of LAWs; LAWs-LAA positive; role of several risk factors in workplace and in life environment.

We also underline the important role of molecular-based allergy in allergic sensitization to furry animals as reported in not-occupational diagnostics [37],[38]. For this reason, we propose to use proteomics in the study of occupational allergies and for LAA in particular [7],[39] to overcome the great variability of LAA prevalence (between 4% and 44%) due to different methodologies and control and preventive measures applied [11],[14]–[16]. In fact, different numbers result on the basis of the definition adopted: Considering IgE positivity response 18% of workers can be defined as LAA, considering answers derived from questionnaire this percentage amounts to 42%, if we consider the combination of IgE positivity response and allergy symptoms the estimate of LAA is 17%. In any case our results appear to be aligned with the literature showing a great variable percentage comprised between 4% and 44% [10]–[16].

Our study was performed with protein microarray and it shows a percentage of 18% of LAWs serum positive to Mus m 1, Mus m 4, Rat n 4 animal allergens which falls within the percentage estimated in literature [10]–[16]; 17 LAWs manifest allergic symptoms whereas only one was asymptomatic, underlying that the role of serological IgE positivity in clinical outcomes. Moreover, our results showed that the occupational exposure is related to manifestation of clinical symptoms in 40% of LAWs with multiple IgE positivity to allergens of mouse and/or rat and 60% with IgE symptomatic.

The study confirmed the onset of symptoms occur in the first three years of work. As reported in the results, 40% of workers (of which 17% had a positive IgE response to mouse and/or rat) referred sneezing, runny and stuffy nose, red and itchy eyes, 15% (of which 10% had a positive IgE response to mouse and/or rat) reported coughing, asthma and wheeze and 13% (of which 4% had a positive IgE response to mouse and/or rat) referred rash and hives.

We retain that using protein microarray is a great opportunity to identify not only susceptibility to occupational allergens (mouse and/or rat Mus m 1, Mus m 4, Rat n 4 animal allergens) but also to evaluate susceptibility to aeroallergens of animal and vegetable, food allergens, and other.

Prevalence of reported symptoms are similar to what is reported in literature, that is a higher percentage for nose and eyes symptoms followed by chest tightness (including asthma) and skin symptoms [10]–[16],[32]. Positivity to indoor, outdoor and animal different from mouse/rat allergens were significantly related to LAWs-LAA positive/sensitized. Risk factors found out in the study are the presence of experienced allergy symptoms (such as asthma and skin symptoms) both in the domestic environment and in the occupational workplace. LAWs-LAA symptomatic have a higher risk than LAWs-LAA positive/sensitized to develop the disease for those working closely with animals (receiving animals, working into the reproduction room, feeding of administering substances, as reported in Table 3). Most reported symptoms were nose and eyes symptoms for 40% workers where 15% reported chest tightness and 13% skin symptoms. Concerning the type of activity carried out with animals, receiving animals or working into the reproduction room or feeding of administering substances are more likely to be a LAWs-LAA symptomatic than LAWs-LAA negative.

The proteomic array for the study of LAA is an innovative approach in the study of environmental and occupational exposure to allergens [7],[39]–[41] and our results strongly suggested the need to further investigate and develop new and increasingly complementary strategies for the management of occupational risk of allergy. It should also be underlined that animal facilities are peculiar environment workplaces characterized by the simultaneous presence of animals (mainly mouse and rat but also rabbit and other species) and humans (women and men) that are altogether exposed to common biological agents and allergens potentially carried by animals to humans and *vice versa*.

## 5. Conclusion

Allergy in the workplace and in animal facilities is a topic extremely timely and necessary to delve into various aspects, risk factors, specific tasks, environmental research and individual sensitization [16],[42],[43].

For this purpose and based on Acts 81/2008 and 26/2014, it is necessary to provide adequate information and communication, through scientific publication, training courses, conferences and other forms of dissemination of the culture of risk prevention [44]–[50] since allergic respiratory diseases are still investigated among workers [51] to deepen the role of co-factors [52].

In this study we obtained a percentage of 18% of LAWs serum positive to rodent allergens; between them 17 LAWs manifest allergic symptoms whereas only one was asymptomatic; the occupational exposure is related to manifestation of clinical symptoms in 40% of LAWs with multiple IgE positivity to allergens of mouse and/or rat and 60% with IgE symptomatic. Our studies are currently in progress and will address the evaluation of the relationship between the sources of environmental air indoor allergens in animal facilities and the susceptibility and immunological profiles of LAWs considering the personal monitoring and the identification of different level risk areas [53],[54] also because *a better understanding of the relationship between exposures and outcome is urgently needed* [42].

The interest towards LAA is currently still present [55],[56] and all prevention actors must encourage an integrated and multidisciplinary approach for the evaluation and management of the laboratory animal allergy.

The scope of the study is to propose a reference methodology to evaluate the IgE response to specific animal allergens. The proposed protein microarray approach has the advantage to provide an *immunological profile* not only for specific occupational allergens but also for a wide variety of environmental and food-borne allergens. A special attention should be paid to sensitized and symptomatic LAWs who may display clinical symptoms and/or the immunological response due to occupational exposure. For these workers more compelling preventive and protective measures as well as a proper health surveillance have to be implemented.
